# Analysis of Balance of Income and Expenditure and Optimal Retirement Age of Pension Insurance Co-Ordination Account Based on Improved Machine Learning Algorithm

**DOI:** 10.1155/2022/5870893

**Published:** 2022-08-31

**Authors:** Shi Yan, Yaodong Zhou, Youlu Zhang

**Affiliations:** School of Economics and Management, Beijing Jiaotong University, Beijing 100044, China

## Abstract

Since the turn of the twenty-first century, the issue of aging has gained international attention. Both developed and developing nations are currently dealing with this issue. To ensure the sustained and healthy growth of the economy and society in the face of an aging society, it is especially important to establish a scientific old-age insurance system and a reasonable retirement system. We are all aware that the key indicators for the state to control the old-age insurance system in the old-age insurance system are the income and expenditure balance of the old-age insurance pooling account and the analysis of the ideal retirement age. In this paper, a better machine algorithm is used. By independently learning the rules present in a large amount of data and gaining new experience and knowledge, machine learning (ML) can increase computer intelligence and give computers decision-making abilities comparable to those of humans. In general, a machine learning algorithm uses the laws it derives from data to predict unknown data after automatically analysing the data. This study's findings suggest that the ideal retirement age and life expectancy are positively correlated, with the ideal retirement age's growth rate 12.57 percent higher than that of life expectancy.

## 1. Introduction

Before the 18th century, the population was mainly concentrated in villages and towns, with poor living environment, underdeveloped medical conditions, diseases, wars, and other reasons, and the population growth was very slow [1]. With the sudden rise of the first industrial revolution, science and technique were born, medical level and grain output were greatly improved, and the global population began to grow slowly. After entering the 19th century, the world ushered in a century-long period of relative peace, and with the help of the second industrial revolution, the population surged, forming the second growth peak in human history [2]. At the same time, due to the declining fertility rate in some developed countries in Europe, some countries began to show signs of aging [3]. Since the 20th century, quite a few countries, including China, have been facing the aging of population. Global challenges have been gradually introduced as aging has spread continuously. The old-age security system is under tremendous strain due to the large population, and the level of contributions and replacements within the system not only affects how well workers will live after they retire but also indirectly affects how dependent retirees will be on old-age insurance [4]. A reasonable contribution rate and substitution rate will help to reduce the financial strain that the aging population is putting on the old-age insurance fund. In addition, a reasonable contribution rate and substitution rate will aid in the establishment and improvement of a multilevel old-age insurance system with Chinese characteristics, even the social security system, as well as in the promotion of the old-age insurance system of governmental institutions and agencies. Therefore, it is important from both a theoretical and practical standpoint to dynamically modify the contribution rate and substitution rate of people and businesses. Government officials and academics have expressed interest in determining a reasonable contribution rate and substitution rate. The income and expenditure of old-age insurance are particularly impacted by the multiple levels and angles of the aging process [5]. Affected by the rapid improvement of aging, the on-the-job workers' support rate for the elderly population has increased, resulting in a decrease in the number of contributors to their pension funds but an increase in the burden. Pension income and expenditure will be unbalanced, which indicates that there will be a gap in the future, and the gap will be bigger and bigger [6]. From a global perspective, the future 21st century will be the century of global population aging. In the degree of aging, there are significant differences among continents in the world [7]. The proportion of the aging population in Asia has remained at a high level for a long time, and the aging growth rate ranks first in the world. The proportion of the elderly population in Europe shows a downward trend; “Silver tsunami” will sweep across most areas; only South Africa will be spared temporarily, but the progress is slow, rather than completely reversing the trend of population aging [8]. Old-age insurance systems in many nations have gradually run into financial difficulties as a result of the rapid increase in the world's aging population. Since their own old-age insurance systems and welfare policies differ from those of the United States, many foreign nations have experimented with adjusting the retirement age and have seen some success [9]. An index that is frequently used to reflect the overall health and quality of life of people in a nation or region is the average life expectancy of the population [10]. The average lifespan of the population has steadily increased, thanks to improvements in living standards, medical and health services, and the infant mortality rate (which has been steadily declining) [11]. Many workers consider the retirement age from their own point of view, and whether it is postponed or not is also highly controversial. For example, people with high education level want to postpone the retirement age in order to give full play to human resources. Many women think that their jobs are not limited by too many physiological conditions and think that they should retire at the same age as men. Some people also think that with the increase of age, their physical condition is not as good as that of their young age, their working passion drops sharply, their working efficiency drops, and it is inappropriate to postpone their retirement age and so on [12]. Undoubtedly, the increasing number of elderly people, the expanding coverage of the pension system, a large number of “empty accounts” running personal accounts, the incipient fiscal deficit, the serious shortage of pension reserves, the gradually obvious “hidden debt,” and the reform cost of the transition between the old and new systems have all increased the financial payment crisis [13]. From this point of view, the pension system is facing huge financial payment pressure in the improvement pattern of “getting old before getting rich” [14].

The innovation of this paper lies in the following:

1. This paper introduces the old-age insurance. Because it is a part of the research topic of this paper, we should discuss it. Part of the pension is the focus of this article, so it was described from the beginning. Scholars generally believe that the basic old-age insurance system is the main component of the modern social security system and an indispensable component. The government adopts laws, regulations, and other means to require enterprises and employees to pay endowment insurance in proportion and give corresponding pensions to workers after their legal retirement to maintain their life after retirement. This policy is the basic endowment insurance system.
2. This article introduces the retirement age. This paper has also studied it, so it is necessary to discuss it. With the rapid spread of population aging, although different countries have implemented different statutory retirement age systems according to their own national conditions, facing this global question, many countries have already or will soon choose to extend their retirement time. The flexible retirement system should be implemented, that is, different people can choose their retirement time independently according to their job nature, health, economic situation, family background, and employment intention, so as to meet the pursuit of retirement life quality.
3. ML can increase computer intelligence by allowing computers to independently learn the rules present in a large amount of data, gain new experience, and learn new things. This allows computers to make decisions that are comparable to those of humans. In order to give computers the ability to learn on their own, ML theory primarily involves designing and examining specific algorithms. In general, ML algorithms use the laws they derive from data to predict unknown data. They automatically analyse the data and derive the laws from it.

## 2. Related Works

Fitzpatrick suggested a complete OLG model idea, modified it, and used it as a vital analysis tool [15]. Lu and Tang suggested the standard to measure economic welfare and the way to improve it and advocated that income distribution should be equalized and the government should play an intervention role in income redistribution [16]. Hatcher suggested to choose ways to increase the contribution rate of individual pension to ensure the adequacy of individual pension accounts [17]. Turner suggested that theory of welfare economics should be based on cardinal utility theory, assuming that consumption can be measured concretely and summed up, also known as “old welfare economics” [18]. A B suggested two OLG models in order to analyse the optimal retirement age better [19]. Yue suggested the combination of unified account and personal account. The purpose of introducing personal account is to accumulate some funds to cope with the peak of retirement, which indicates that the old-age insurance system has achieved a major breakthrough of the “partial accumulation system” [20]. Chen suggested the basic idea of “small step by step,” but the reform opportunity, rhythm grasp, the path of delaying retirement, and the optimal retirement age of different social groups have all become difficult questions to be solved in the policy reform of delaying retirement [21]. Den et al. suggested two options of combining social pooling with individual accounts, allowing them to choose according to the actual situation and even modifying them appropriately [22]. Meng et al. suggested a two-stage reform plan: first, the retirement age of women should be adjusted to 55 without distinction. Then, men will retire at the age of 65 at the rate of 1 year/6 years and women at the rate of twice that of men [23]. Steiber and Kohli suggested a new idea of supplementing enterprise insurance and personal savings endowment insurance, but overall, it is still the product of the times under the planned economy system [24].

With the advancement of science and technology, ongoing economic growth, ongoing medical and health conditions improvement, and a significant rise in life expectancy, the population aging trend is becoming more and more obvious, and the global question of how to balance the supply and demand of crisis endowment insurance funds has emerged. To varying degrees, every nation in the world is improving and reforming its endowment insurance system. Old-age insurance is a type of insurance designed to reduce the risk that an elderly person's income will be interrupted or reduced, which will result in a decline in living standards. Endowment insurance plays a crucial role in China's national economy and social advancement as a crucial component of the social system. The need to restructure and enhance the old-age insurance system is urgent. The old-age insurance reform is currently in a phase of transition, with the goal of implementing the changeover between the old and new systems. The analysis of the balance of payments and the ideal retirement age for the pension insurance pooling account, which is of great significance, is based on an improved machine learning algorithm in this paper.

## 3. Endowment Insurance and Optimal Retirement Age

### 3.1. Endowment Insurance

The disparity between public pension revenue and expenditure in various nations is getting progressively worse as the proportion of the elderly population rises. The operating mechanism of endowment insurance systems is the same across all countries, despite the variations in system types. Most academics concur that the fundamental old-age insurance system is both the main and most essential part of the contemporary social security system. The government adopts laws, regulations, and other means to require enterprises and employees to pay endowment insurance in proportion and give corresponding pensions to workers after their legal retirement to maintain their life after retirement. This policy is the basic endowment insurance system.

Let us say *S*(*t*) is a discrete and discontinuous function, but the increase or decrease of personal normal savings is relatively small compared with the total personal savings, especially after working for a period of time. In order to study the method, *S*(*t*) is regarded as continuously differentiable's function , which does not affect its economic and practical significance, so it can be studied by the differential method.

Set the time interval of [*t*, *t*+Δ*t*], of which Δ*t* is as small as possible. The increase (decrease) of personal savings is

(1) $$\begin{array}{c} S\left(t+\Delta t\right)-S\left(t\right)=\frac{\Delta S\left(t\right)}{\Delta t}\Delta t. \end{array}$$

Let *r*(*t*) be the risk-free interest rate at *t* hours (replaced by the bank interest rate), *α* be the wage growth rate, *u*_1_(*t*) be the personal contribution rate, *β* be the consumption change rate, *q*(*t*) be the replacement rate of savings replaced by personal accounts, *X*_1_(*t*) be the personal consumption at *t* hours, and *I*(*t*) be the personal account pension at *t* hours. Assuming that wage increases, personal pension contribution increase and consumption increase are proportional to wage *W*(*t*) and time Δ*t*, and there are

(2) $$\begin{array}{c} \frac{\Delta S\left(t\right)}{\Delta t}\cdot \Delta t=\left(r\left(t\right)-q\left(t\right)\right)S\left(t\right)\Delta t+\alpha \left(1-u_{1}\left(t\right)\right)W\left(t\right)\Delta t-\beta X_{1}\left(t\right)\Delta t. \end{array}$$

When Δ*t*⟶0, *S*(*t*) satisfies the differential equation,

(3) $$\begin{array}{c} \frac{dS\left(t\right)}{dt}=\left(r\left(t\right)-q\left(t\right)\right)S\left(t\right)+\alpha \left(1-u_{1}\left(t\right)\right)W\left(t\right)-\beta X_{1}\left(t\right). \end{array}$$

On the other hand, wages are distributed among consumption, savings, and personal account pensions:

(4) $$\begin{array}{c} X_{1}\left(t\right)=W\left(t\right)-I\left(t\right)-S\left(t\right). \end{array}$$

Take derivatives of *t* on both the sides, and there are

(5) $$\begin{array}{c} \frac{\text{d}X_{1}\left(t\right)}{\text{d}t}=\frac{\text{d}W\left(t\right)}{\text{d}t}-\frac{\text{d}I\left(t\right)}{\text{d}t}-\frac{\text{d}S\left(t\right)}{\text{d}t}. \end{array}$$

For retirement consumption, assume the individual's consumption at *τ* hours in the retirement period is *X*_2_(*τ*), and there are

(6) $$\begin{array}{c} X_{2}\left(\tau \right)=u_{3}\left(\tau \right)\cdot W\left(\tau \right)+\frac{\left(1+r_{1}\right)S_{I_{1}}}{T-t_{1}}+\frac{\left(1+r_{1}\right)I_{I_{1}}}{T-t_{1}}. \end{array}$$

Among them, *S*_*I*_1__ is the total amount of savings during the working period, *I*_*t*_1__ is the total amount of personal account principal during the working period, *r*_1_ is the storage interest rate during the whole working period, and *u*_3_(*τ*) is the replacement rate at *τ* hours.

The greatest utility of the basic old-age insurance is to maintain the life of retired workers. Its implementation is beneficial to the society and individuals. Its main contents are as follows: it helps to strengthen the promotion of total social productivity, mobilize the participation enthusiasm of individual workers, and continuously provide the required labor supply for social reproduction. Delaying retirement can alleviate the financial pressure of pension to a certain extent, but it cannot fundamentally solve the account gap of pension, and there may be a pension deficit question in the future. There is no inevitable connection between the pension gap and the aging of the population, and there is great uncertainty about whether delaying retirement can make up for the pension gap. Although delaying retirement can achieve the effect of increasing income, the insured can receive more pensions in a longer life, which may offset the degradation effect of delaying retirement policy on the pension deficit. The number of employees participating in the basic old-age insurance system has been increasing, and their basic old-age insurance income has been increasing, and the total pension is gradually increasing, but the growth rate has slowed down. The appearance of prosperity cannot hide the hidden crisis of basic old-age insurance.

### 3.2. Optimal Retirement Age

If delaying retirement is an effective plan to solve China's pension dilemma at present, how should the best time for reform be determined? With the rapid spread of population aging, although different countries have implemented different statutory retirement age systems according to their own national conditions, facing this global question, many countries have already or will soon choose to extend their retirement time. Some scholars believe that the formulation and implementation of the delayed retirement policy cannot be rushed for a while, but it needs to wait until the pressure of old-age care becomes increasingly urgent, and the employment pressure is not obviously aggravated.

Considering that the policy of delaying retirement involves a wide range of issues and there are big differences of opinions, it is inevitable that there will be many obstacles in its implementation, and it is impossible to achieve it in one step. Therefore, most of the schemes are designed with flexible adjustment and gradual steps, so as to minimize the adverse effects caused by the reform and consider social fairness and efficiency to a greater extent. First of all, there is a mismatch between the rising life expectancy and the stagnant retirement system. Second, the education level of residents is gradually rising, and the number of years of education continues to increase, resulting in the working hours of school-age workers shrinking. Finally, in order to maintain the domestic basic pension balance, it is possible to increase the overall social pension payment rate of enterprises, which will inevitably reduce the interests of enterprises, weaken the competitiveness of the enterprises where they are located, and stabilize the average pension replacement rate.

From the international experience, most developed countries adopt a gradual and steady reform scheme based on the actual age structure of the population, which is easy for the society to digest gradually and reduce the resistance to reform. It is best to avoid raising the contribution rate of social endowment insurance for enterprises and extending the retirement age may be a more reasonable way. Some scholars also believe that the flexible retirement system should be implemented, that is, different groups of people can choose their retirement time independently according to their job nature, health, economic status, family background, and employment intention, in order to meet the pursuit of retirement life quality. The flexible retirement policy is more recognized because it makes the labor market more flexible, and at the same time, it is conducive to the optimization of individual resource allocation and the stability of the economic environment.

## 4. ML and Model Building

### 4.1. ML

**Figure 1:**
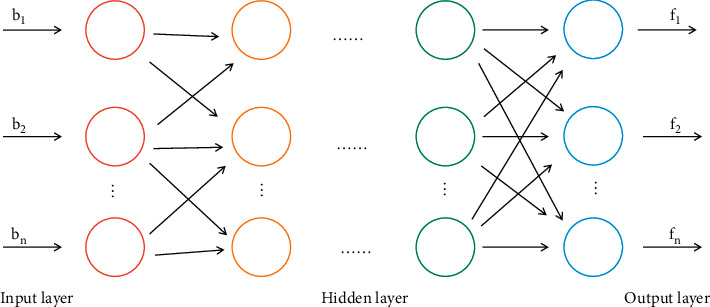
Classical neural network model.

As science and technology have advanced, artificial intelligence techniques have been used more and more, and machine learning (ML) has always been the subject of people's attention as its primary component. This article provides a definition of Hadoop based on ongoing research into the technology: To have a large number of high-growth message assets with better process optimization and discovery capabilities, Hadoop needs a new processing mode. The practical use of data mining techniques can advance social science and methodology, enhance people's capacity for message processing, and be extremely important in the information age. Hadoop has clear examples of the 4 Vs, which stand for low value density, large data capacity, diverse data types, and quick data processing. Previously, the traditional data mining algorithm involved optimising the ML algorithm using the dataset. It has been challenging for this traditional ML method to meet the demand for data mining in the current large amount of heterogeneous data due to the current aspects of collection, retrieval, storage, sharing, analysis, and processing. Learning is an essential human skill, and as technology has advanced, computers are now capable of gradually learning new things. The classical neural network model is shown in Figure 1.

One goal of learning enhancement is to obtain reward function. Reward function is usually a scalar, which is an evaluation of the concrete behavior of an entity. The general reward title is set to a positive number, whereas the scalar of punishment is negative. Reward functions are usually described as the following function:

(7) $$\begin{array}{c} R_{t}=r_{t+1}+\gamma r_{t+2}+\gamma^{2}r_{t+3}+\ldots =\sum_{k=0}^{\infty }\gamma^{k}r_{t+k+1}. \end{array}$$

The gathering of parameters *γ* has a great influence on the convergence speed of the algorithm. Generally speaking, the parameter *γ* takes a value between 0 and 1.

*T*  *D*(0): The update formula of algorithm value function is as follows:

(8) $$\begin{array}{c} V\left(S_{t}\right)\leftarrow V\left(S_{t}\right)+\alpha \left[r_{t+1}+\gamma V\left(S_{t+1}\right)-V\left(S_{t}\right)\right], \end{array}$$

where *α* represents the learning factor, *γ* represents the discount rate, *V*(*S*_*t*_) represents the value function of the entity at time *t* and state *S*_*t*_, *V*(*S*_*t*+1_) represents the predicted state value function of the entity at time *t*+1 and state *S*_*t*+1_, and *r*_*t*+1_ represents the instantaneous reward value obtained after the entity transitions from state *S*_*t*_ to state *S*_*t*+1_ after performing the behavior.

In order to improve its convergence speed, the *T*  *D*(*λ*) algorithm is developed, which can roll back the instant reward value obtained by the entity by any step. *T*  *D*(*λ*): The iterative formula of the algorithm is as follows:

(9) $$\begin{array}{c} V\left(S_{t}\right)\leftarrow V\left(S_{t}\right)+\alpha \left[r_{t+1}+\gamma V\left(S_{t+1}\right)-V\left(S_{t}\right)\right]e\left(S\right), \end{array}$$

where *e*(*S*) indicates the degree of election in the state *s*, and *λ* represents a number between 0 and 1. When *λ*=0, the algorithm degenerates to *T*  *D*(0).

(10) $$\begin{array}{c} e\left(S\right)=\left\{\begin{array}{cc} \gamma \lambda e\left(S\right)+1, & \text{if }s\text{ is current state},\\ \gamma \lambda e\left(S\right), & \text{otherwise}. \end{array}\right.. \end{array}$$

What is ML? By independently learning the rules present in a large amount of data and gaining new experience and knowledge, ML can increase computer intelligence and give computers decision-making abilities comparable to those of humans. Designing and examining various algorithms that give computers the ability to learn on their own is the main focus of machine learning theory. In general, a machine learning algorithm uses the laws it derives from data to predict unknown data after automatically analysing the data. In reality, data mining's limited data processing ability makes it more and more challenging to analyse data. Due to its interdisciplinary nature, machine learning algorithms are able to simulate human behavior and automatically pick up new skills and knowledge. The ability to analyse data can be significantly improved by applying ML algorithms to data mining. People can learn and imitate well, but the process of learning is very difficult. ML theory underlies this alleged procedure. The practise of using computer simulation techniques to study how humans learn, innovate on previously held knowledge, enhance analysis, and find answers to problems is known as machine learning (ML).

The main goal of machine learning is to gain knowledge through extensive data analysis. The Hadoop technique has attracted the attention of an increasing number of experts in recent years. ML has developed quickly, thanks to the advancements in hardware technology, Hadoop's computing power, and storage capacity. The ability of computers and human brains to answer questions is still very different at this point in machine learning's development, which is still in its early stages. The ML technique is the main source for the study of learning mechanisms. In the current Hadoop environment, data analysis has drawn attention from a variety of industries. ML can absorb knowledge more quickly, which can successfully encourage the advancement of the machine technique. In the field of ML, reinforcement learning is a crucial algorithm. The reinforcement algorithm has the advantage of essentially not requiring prior knowledge of the environment as it learns the best course of action for the dynamic system under consideration based on perception of the surrounding environment and primarily corrects its own behavior strategy through trial and error.

With regard to ML, there are two main research directions in its improvement: the first is the study of the learning mechanism, which pays more attention to the exploration of human learning mechanism simulation. The second is the study of effective use of message, which focuses on finding and discovering valuable and cognizable potential knowledge from huge databases. The research direction of learning mechanism originated from the ML technique. With the advent of Hadoop environment, there is a strong demand for data analysis in all walks of life. ML can acquire knowledge more quickly, which makes ML become a booster for the improvement of the machine technique. The main purpose of ML is to get corresponding conclusions from data independently. The ML algorithm is mainly the process of solving the optimal solution of a question by mathematical and statistical methods. In the current Hadoop environment, how to adopt effective learning methods is the significance of ML at present, and ML will also become a widely respected and popular learning and service technique.

### 4.2. Model Building

**Figure 2:**
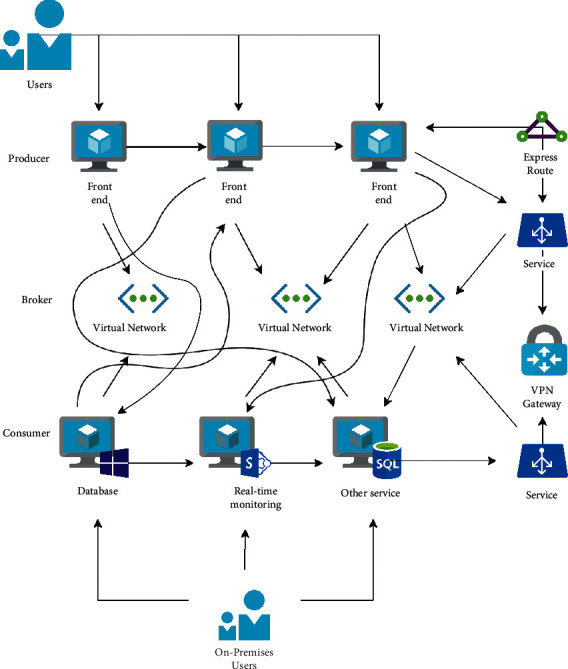
Architecture diagram of the endowment insurance system.

A country or society typically creates the old-age insurance system through mandatory legislation in accordance with its unique national circumstances. Workers will be guaranteed a minimal standard of living if they leave their jobs or reach the legal age for which they are required to perform compulsory labor, as specified by the applicable laws. There are two fundamental market-based pension models: one involves providing home care for the elderly and the other involves enrolling in a social pension institution. They both share service content that is market-oriented and socialised to start. Second, there are some differences between them in terms of how old-age resources are provided and how old-age care is provided. Urban employees' basic endowment insurance fund is split into individual and overall accounts. The structure diagram of the old-age insurance system is shown in Figure 2.

**Figure 3:**
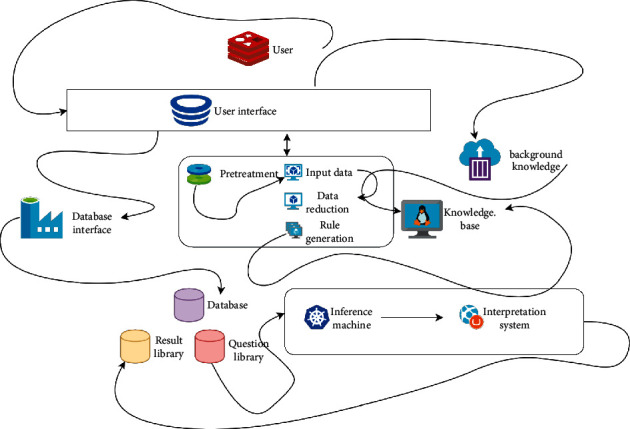
Structure of the pension system.

The overall account fund implements the pay-as-you-go system, that is, it pays retirees' pensions with the endowment insurance premiums paid by on-the-job employees in the same period, which has the function of income redistribution between generations. However, the personal account fund implements the full accumulation system, that is, individuals accumulate funds by saving during the whole employment period, and after retirement, they get all the interest income of the fund, which does not have the function of income redistribution between generations. The pension system structure is shown in Figure 3.

Social pooling specifically refers to the fact that in the same period, the pension fees paid by workers will be used for the pension collection of the retired elderly, which will be explained by the formula in the subsequent government departments. Moreover, considering that the source of social pooling is paid by enterprises, social pension pooling will not be included in individual behavior, but in corporate behavior.

Assuming that the salary of employees in the first year of employment is *w*, the annual growth rate of individual wages is *g*, the individual contribution rate is *c*(*c*=8%), the employment period is *m*(*m* ≥ 15), the predetermined interest rate of individual account is *i*(*i* ≠ *g*), the average life expectancy is *l*, and the average retirement age is *r* and the payment time is at the beginning of each year, then the final value of each year's contribution at the end of employment is as follows.

Final value from the beginning of the first year to the end of *m*: *cw*(1+*i*)^*m*^.

Final value from the beginning of the second year to the end of *m*: *cw*(1+*g*)(1+*i*)^*m*−1^.

……

Final value from the beginning of *m* to the end of *m*: *cw*(1+*g*)^*m*−1^(1+*i*):

(11) $$\begin{array}{c} S=cw{\left(1+i\right)}^{m}+cw\left(1+g\right){\left(1+i\right)}^{m-1}+\text{…}+cw{\left(1+g\right)}^{m-1}\left(1+i\right)\\ =cw\left(1+i\right)\frac{{\left(1+i\right)}^{m}-{\left(1+g\right)}^{m}}{i-g}. \end{array}$$

The salary of an employee in the year before retirement can be expressed as *w*(1 − *g*)^*m*−1^.

By

(12) $$\begin{array}{c} c=8\%,b=\frac{s}{\left(l-r\right)}. \end{array}$$

The replacement rate of personal account pension is

(13) $$\begin{array}{c} T=\frac{b}{w{\left(1+g\right)}^{m-1}}=\frac{S/l-r}{w{\left(1+g\right)}^{m-1}}=\frac{0.08w\left(1+i\right){\left(1+i\right)}^{m}-{\left(1+g\right)}^{m}/i-g}{\left(l-r\right)w{\left(1+g\right)}^{m-1}}\\ =\frac{0.08\left(1+i\right)}{l-r}\frac{\left[{\left(1+i\right)}^{m}-{\left(1+g\right)}^{m}\right]}{{\left(1+g\right)}^{m-1}\left(i-g\right)}=0.08\left(1+i\right)\frac{1}{\left(i-g\right)\left(l-r\right)}\left[\left({\left(\frac{1+i}{1+g}\right)}^{m-1}\left(1+i\right)-\left(1+g\right)\right)\right]. \end{array}$$

**Figure 4:**
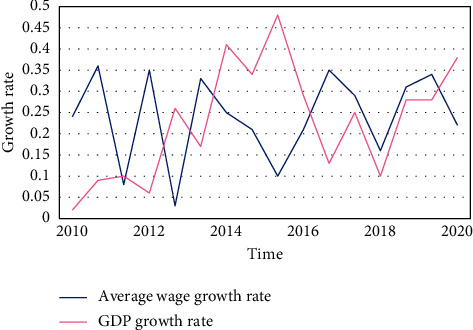
Trends of GDP growth rate and average wage growth rate.

**Figure 5:**
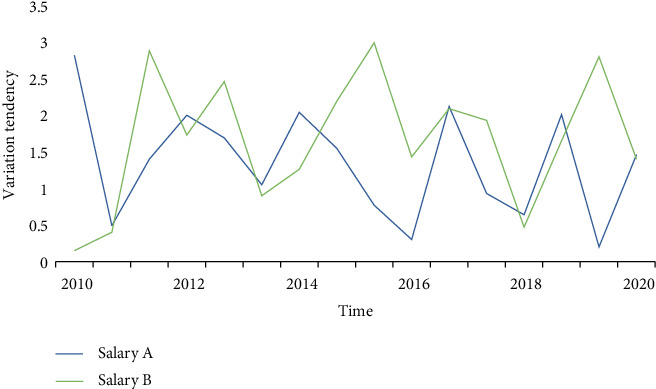
Salary scatter chart.

**Figure 6:**
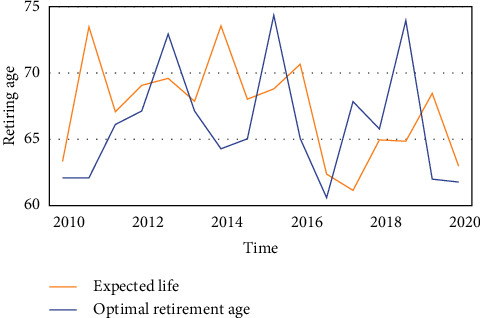
Comparison of optimal retirement age.

The goal of home-based care for the elderly is to mobilize all social forces and create a home-based care system that is most in line with the preferences of the elderly, places the family at its center, is supported by the community care service network, and is insured by the old-age insurance system. However, the institutional pension is entirely provided by society in terms of the funding source, the nature of the service, and the method of operation. This means that the financial support and life services for the elderly in their later years are provided by society, including pension, medical expenses, welfare expenses, relief expenses, and life care for the elderly, which are provided by social security institutions, governments at all levels, businesses, and institutions. Figures 4, 5, and 6 show how the continuous rise in life expectancy results in a more serious aging situation. The ideal retirement age and life expectancy are positively correlated, with the ideal retirement age growing at a rate 12.57 percent higher than life expectancy.

**Figure 7:**
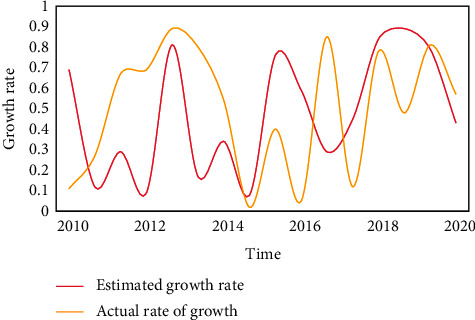
Population growth rate under the two-child policy.

**Figure 8:**
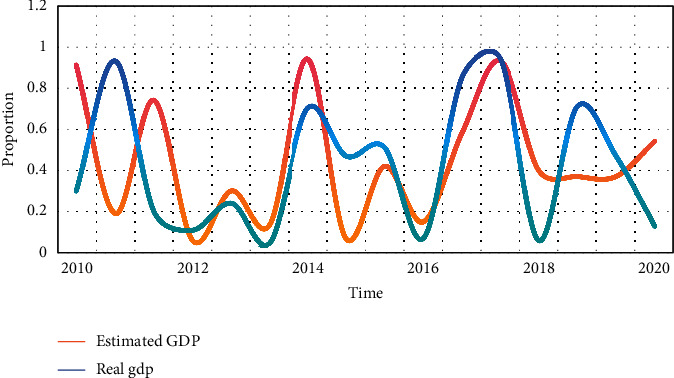
GDP ratio comparison.

**Figure 9:**
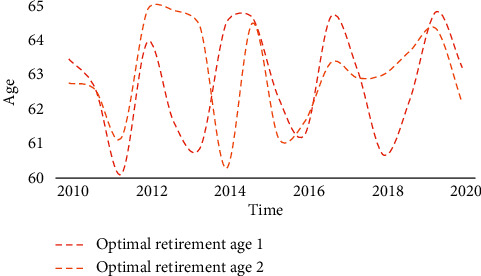
Simulation of optimal retirement age.

Living forms include nursing homes, nursing homes, and care for the elderly. It is characterized by centralized pension. The old-age insurance system is mainly about the management and operation of pensions, which are all based on mathematical knowledge and operated through actuarial management. The old-age insurance system is a comprehensive subject integrating economy, society, and management, involving many vital concepts. The construction of social welfare function is mainly based on the maximization of social welfare. As a policy maker, the government should draw up a retirement system with reference to this premise. The basic old-age insurance adopts the mode of unified accounting, which forms the “hidden debt” of the old-age insurance for various historical reasons. From Tables 1, 2, and 3 and Figures 7, 8 and 9, it can be seen that although the implementation of the two-child policy has improved the total fertility rate as a whole, the proportion of women of childbearing age continues to decline and China's population growth rate will slow down in the future. At the same time, the changing trend of the optimal retirement age is consistent with the population growth rate, so the optimal retirement age is positively related to the population growth rate.

In order to make up for the gap of endowment insurance fund, the government misappropriated the fund of individual account, resulting in serious deficit of individual account and a series of “mixed accounts” questions, which caused great concern of the state and provinces and cities. The principle of raising pension funds must follow the balance between collection and payment; in other words, the raising of pension funds should keep the basic financial balance with the payment of pension expenses according to regulations.

## 5. Conclusions

Many nations run the risk of widening the gap between income and pension expenditures as a result of the population's rapid aging. The average wage growth rate is a factor in both the income and expenditure models of the overall pension account. Raising the wage growth rate will raise the pension level for retirees while also raising the payment standard for employees who are still working. However, because the pension benefits received by retirees are only increased to the extent that the wages of the on-the-job employees have increased, after raising the average wage growth rate, the increased income of the pension fund is naturally higher than the expenditure, which not only significantly lessens the pressure on the pension fund's ability to make payments but also serves as an obvious buffer during the implementation of the deferred retirement policy. The ideal retirement age is a dynamic equilibrium age that depends on a variety of factors rather than being a fixed age. People who started working later and those who saved less early on tend to retire later, given the assumption that the ideal retirement age is earlier than the voluntary retirement age. This study's findings suggest that the ideal retirement age and life expectancy are positively correlated, with the ideal retirement age's growth rate 12.57 percent higher than that of life expectancy.

## Figures and Tables

**Table 1:** Population growth rate under the two-child policy.

	2010	2012	2014	2016	2018	2020
Estimated growth rate	0.69	0.09	0.34	0.59	0.84	0.43
Actual rate of growth	0.11	0.69	0.54	0.05	0.78	0.57

**Table 2:** GDP ratio comparison.

	2010	2012	2014	2016	2018	2020
Estimated GDP	0.91	0.06	0.94	0.15	0.4	0.54
Real GDP	0.3	0.11	0.7	0.07	0.06	0.13

**Table 3:** Simulation of optimal retirement age.

	2010	2012	2014	2016	2018	2020
Optimal retirement age 1	63.46	63.96	64.5	61.27	60.67	63.21
Optimal retirement age 2	62.76	64.89	60.28	61.65	63.01	62.17

## Data Availability

The data used to support the findings of this study are available from the corresponding author upon request.

## References

[1] (2018). The health implications of social pensions: evidence from China’s new rural pension scheme[J]. *Journal of Comparative Economics*.

[2] Menoncin F., Vigna E. (2017). Mean–variance target-based optimisation for defined contribution pension schemes in a stochastic framework. *Insurance: Mathematics and Economics*.

[3] Knell M. (2018). Increasing life expectancy and NDC pension systems. *Journal of Pension Economics and Finance*.

[4] Gouveia A. F. (2017). Political support for reforms of the pension system: two experiments. *Journal of Pension Economics and Finance*.

[5] Abínzano I., Muga L., Santamaría R. (2017). Bad company. The indirect effect of differences in corporate governance in the pension plan industry. *International Review of Financial Analysis*.

[6] Cheng Y., Li C., Johnston L. A. (2018). The intergenerational education spillovers of pension reform in China. *Journal of Population Economics*.

[7] Brzeszczynski J., Bohl M. T., Serwa D. (2019). Pension funds, large capital inflows and stock returns in a thin market. *Journal of Pension Economics and Finance*.

[8] Blake D., Sarno L., Zinna G. (2017). The market for lemmings: the herding behavior of pension funds. *Journal of Financial Markets*.

[9] Chen D. H., Beetsma R. M., Broeders D. W. G. A., Pelsser A. A (2017). Sustainability of participation in collective pension schemes: an option pricing approach. *Insurance: Mathematics and Economics*.

[10] Chen Z., Li Z., Zeng Y., Sun J. (2017). Asset allocation under loss aversion and minimum performance constraint in a DC pension plan with inflation risk. *Insurance: Mathematics and Economics*.

[11] Kreiner C. T., Leth-Petersen S., Skov P. E. (2017). Pension saving responses to anticipated tax changes: evidence from monthly pension contribution records. *Economics Letters*.

[12] Naumann E. (2017). Do increasing reform pressures change welfare state attitudes? An experimental study on population ageing, pension reform preferences, political knowledge and ideology. *Ageing and Society*.

[13] Tang M. L., Chen S. N., Lai G. C., Wu T. P. (2018). Asset allocation for a DC pension fund under stochastic interest rates and inflation-protected guarantee. *Insurance: Mathematics and Economics*.

[14] Kerner A. (2018). Pension returns and popular support for neoliberalism in post-pension reform Latin America. *British Journal of Physical Education*.

[15] Fitzpatrick M. D. (2017). Pension-spiking, free-riding, and the effects of pension reform on teachers’ earnings. *Journal of Public Economics*.

[16] Lu T. J., Tang N. (2019). Social interactions in asset allocation decisions: e. *Journal of Economic Behavior & Organization*.

[17] Hatcher M. (2019). Should a pension reform be announced? A reply[J]. *Economics Letters*.

[18] Turner J. A., Hughes G., Chlon-Dominczak A. (2018). Improving pension income and reducing poverty at advanced older ages: longevity insurance benefits in Ireland and Poland as models for the United States. *Journal of Portfolio Management*.

[19] Bertoni M., Brunello G., Mazzarella G. (2018). Does postponing minimum retirement age improve healthy behaviors before retirement? Evidence from middle-aged Italian workers - ScienceDirect. *Journal of Health Economics*.

[20] Yue L. (2018). Paradoxical effects of increasing the normal retirement age: a prospective evaluation - ScienceDirect. *European Economic Review*.

[21] Chen H. J. (2018). Fertility, retirement age, and pay-as-you-go pensions﹜ouゞo pensions. *Journal of Public Economic Theory*.

[22] Den B., Zijderveld S. A., Bruers J. (2018). Preferred and actual retirement age of oral and maxillofacial surgeons aged 55 and older in The Netherlands: a longitudinal study from 2003 to 2016. *Human Resources for Health*.

[23] Meng A., Nexø M. A., Borg V. (2017). The impact of retirement on age related cognitive decline – a systematic review. *BMC Geriatrics*.

[24] Steiber N., Kohli M. (2017). You can’t always get what you want: actual and preferred ages of retirement in Europe. *Ageing and Society*.

